# Does an Isoniazid Prophylaxis Register Improve Tuberculosis Contact Management in South African Children?

**DOI:** 10.1371/journal.pone.0080803

**Published:** 2013-12-10

**Authors:** Nelda van Soelen, Karen du Preez, Susan S. van Wyk, Anna M. Mandalakas, Don A. Enarson, Anthony J. Reid, Anneke C. Hesseling

**Affiliations:** 1 Desmond Tutu TB Centre, Department of Paediatrics and Child Health, Faculty of Medicine and Health Sciences, Stellenbosch University, Cape Town, Western Cape, South Africa; 2 Section on Retrovirology and Global Health, Department of Pediatrics, Baylor College of Medicine, Houston, Texas, United States of America; 3 The Tuberculosis Initiative, Texas Children’s Hospital, Houston, Texas, United States of America; 4 The International Union Against Tuberculosis and Lung Disease, Paris, France; 5 Operational Research Unit, Médecins Sans Frontières Operational Centre Brussels, Luxembourg; London School of Hygiene and Tropical Medicine, United Kingdom

## Abstract

**Setting:**

We compared the change in child household contact management of pulmonary tuberculosis (TB) cases before and after the implementation of an isoniazid preventive therapy (IPT) register in an urban clinic setting in Cape Town, South Africa.

**Objectives:**

We determined if the presence of an IPT register was associated with an increase in the number of child contacts identified per infectious case and the proportion of identified children who were started on IPT.

**Design:**

We reviewed routine programme data on IPT delivery to children during two time periods (May 2008–October 2008 and May 2011–October 2011), before and after the implementation of an IPT register used by routine clinic personnel.

**Results:**

Adult TB case demographic and clinical characteristics from the two observation periods were similar. During the post-register period, more child contacts per adult case were identified (0.7 (54 children) vs. 0.3 (24 children)), more of the identified children were started on IPT (54 vs. 4) and 37% of those who started, completed six months of treatment compared to the pre-register period where no adherence information was recorded.

**Conclusions:**

After pilot implementation of an IPT register, documented identification of child contacts, IPT initiation and IPT adherence documentation in TB exposed children was improved. Our findings support further exploration of the potential impact of using standardised IPT recording and reporting in routine clinics in high-burden TB settings to improve TB prevention efforts targeted at young children. Future efforts to improve IPT delivery should be systematic and comprehensive in order to support a change in current operational IPT delivery practices in TB programs.

## Introduction

Children constituted an estimated 6% (490 000) of the total number of tuberculosis (TB) incident cases globally in 2011 against a background of considerable under-reporting of TB in children [1]. Isoniazid preventive therapy (IPT) is a safe and cost-effective intervention [2] that can prevent TB in as much as 60% of individuals infected with *Mycobacterium tuberculosis* (*M.tb*) [3]. Given the high rate of paediatric TB in especially high-burden settings such as South Africa (child TB notification rate of 671 per 100 000 comprising 17% of the total TB caseload (unpublished data, Western Cape Department of Health 2008)), the failure to initiate preventive Isoniazid therapy in child contacts represents a significant missed opportunity for TB prevention in children [10]. This is especially true for young children since up to 50% of infants (≤12 months of age) will progress to TB disease following infection with *M.tb*, in the absence of IPT [11].

In line with the evidence of the benefits of IPT, the South African National TB Programme (SANTP) guidelines, consistent with the World Health Organization (WHO), recommend that all children under five years of age [4] and all HIV-infected children, regardless of age, receive IPT if they are exposed to a household adult with bacteriologically confirmed TB (sputum smear- and/or culture positive pulmonary TB), once active TB disease is excluded [5]. Despite these official prevention guidelines, previous studies from Cape Town South Africa [6], [7] and other high-burden settings [8], [9], [10], have illustrated the challenges of documenting contacts in routine clinical records and indicated that only a low proportion of eligible identified contacts received IPT in these high-burden settings.

In order to address this issue, a standardised register tool to monitor IPT delivery in children has previously been recommended as an operational tool to assist IPT delivery by the WHO [11]. However to our knowledge this approach has not been systematically evaluated and documented in an African setting. To address this implementation gap, we introduced an IPT register based on this recommendation in a clinic in a high-TB burden community in Cape Town, South Africa. We used this opportunity to evaluate if the introduction of an IPT register was associated with an increase in a) the number of child household contacts identified per infectious adult TB case and b) the proportion of identified children started on IPT.

## Method

### Design

This operational study evaluated routine programme data on IPT delivery in children during two time periods (May 2008–October 2008 and May 2011–October 2011), before and after the implementation of an IPT register that was used by routine TB service personnel (Figure S1).

### Setting

The study was conducted in a clinic in Cape Town South Africa, which delivers comprehensive health care including TB services, free of charge. The clinic serves a stable population (24 483, 2001 SA Census data) and is characterized by low socio-economic status, high TB burden (>900 per 100 000 in 2008), and relatively low HIV prevalence (16% amongst adult TB cases in 2008 (unpublished data, Western Cape Department of Health)). One quarter of the population is estimated to be less than 13 years of age and retreatment cases accounted for approximately a third of adult cases (unpublished data, Western Cape Department of Health). While residents have access to private health services, the majority seek primary care at public health services in the community.

### Sample

The study consecutively selected bacteriologically-confirmed (sputum smear- and/or culture positive) adult cases recorded in the local TB treatment registers during the defined study periods. Case records were examined to identify child contacts (under five years of age) living at the same household address as the TB case. The study sample included all the child contacts identified by the adult chart review and those who were recorded in the IPT register (post-register period only) by routine health care personnel in the TB treatment room.

### IPT Protocol

TB clinic personnel offered IPT to all identified child contacts of a bacteriologically confirmed TB case. The algorithm in use at the time of the pre-register review required that all TB-exposed children receive formal TB screening (chest radiograph and tuberculin skin test) prior to initiating IPT. Prior to implementation of the IPT register, this algorithm was changed, to require TB screening for symptomatic child contacts only (i.e. coughing, failure to thrive, fever, lethargy, unwell), and IPT initiation in the remainder, as per the 2006 WHO guidance for high-burden settings.

### Data Collection

Data from the pre-register period (May – October 2008) had been previously extracted from the records of cases identified in the TB registers from the clinic [7], using a standard data extraction tool to collect the following information: adult TB case demographics and disease characteristics, documented child contact demographic data and child TB disease and IPT status. Information on child contacts in the pre-register period was collected from the following sources, where available: general clinic folders, clinic TB folders, and any additional medical records identified.

**Figure 1:**
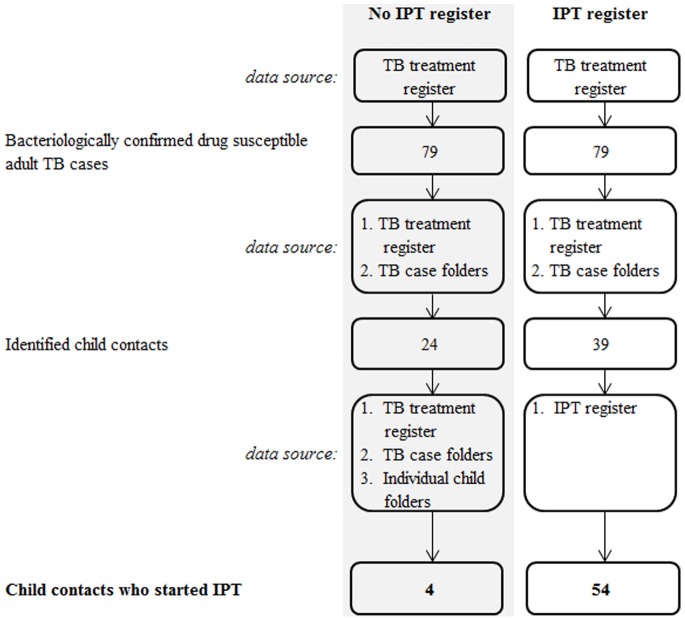
Flow diagram describing the identification of children documented as eligible for IPT and started on IPT during the pre and post-register study periods.

Before the IPT register was implemented, data on IPT delivery to children was only available from case record reviews, without standardised recording. Following the implementation of the register, IPT uptake data were available directly from the register and included: child name, age, sex, residential address and details of IPT administration, linked to the adult cases in the TB treatment register. Additional data for the post-register period (May – October 2011) were collected from the clinic using data extraction tools similar to those used during the pre-register period in order to link the children recorded in the IPT register to the relevant adults in the TB treatment register [7]. All IPT delivery data presented from the post-register period therefore originated from the IPT register (Figure 1).

### Outcome Measures

The outcomes of interest were a) the number of identified household child contacts <5 years of age per adult case identified and b) the proportion of these children documented to have been initiated on IPT at the clinic. The main determinant was the study period, prior to, or after, the implementation of the IPT register.

### Analysis

Following dual data entry into EpiData and data validation, analysis was performed using STATA/IC version 12.1. Continuous variables were described by mean and standard deviation and categorical variables were described with frequencies and proportions. The STROBE guidelines [12] for observational research were followed for reporting purposes.

### Ethics

The study was approved by the Stellenbosch University Ethics Board, the Western Cape Department of Health in Cape Town, South Africa and The Union’s Ethics Advisory Group, The Union, Paris, France. Individual informed consent was not required as the data were collected from routine data that were analysed anonymously.

## Results

Previously reported results from the pre-register period at the clinic identified 100 consecutive adult pulmonary TB cases of which 79% (n = 79) were bacteriologically confirmed. The gender of cases was evenly distributed and the mean age was 35 years. In the post-register period, there were 96 consecutive adult TB cases, of which 82% (n = 79) had bacteriologically confirmed pulmonary TB. Gender was equally represented with a mean age of 39 years, notably a high proportion of adults were classified as retreatment cases (35%, n = 28) and 12% (n = 7) were HIV- infected. The HIV status (positive or negative) amongst adult TB cases was recorded for 72% of cases during the post-register period, in contrast with 97% from the pre-register period [7] (Table 1).

**Table 1:** Demographic details of eligible adult tuberculosis cases prior to and after implementation of an IPT register in a clinic in Cape Town, South Africa.

	Total cases^1^	Total cases^1^
	April 2008–October 2008	May 2011–October 2011
	n = 79 (%)	n = 79 (%)
Age (years; mean ± SD)	35±12	39±13
Male gender	49 (62)	38 (48)
HIV infected	13 (16)^2^	7 (12)^3^
Retreatment episode	27 (34)	28 (35)

SD = standard deviation.

1 Bacteriologically confirmed and not on a drug-resistant treatment regimen.

2 3% of HIV results were unknown overall.

3 HIV results only available for 57 adults.

Following the implementation of the IPT register, there were more child contacts per adult source case identified [0.7 (54 children) vs. 0.3 (24 children), Table 2]. Prior to the register implementation, only four of the identified IPT-eligible children had been started on IPT and there was no documentation identified that could inform about treatment adherence. In the presence of the IPT register, 15 more children were identified and started on IPT according to the IPT register in addition to the 39 children recorded as eligible for IPT in the TB register and TB case folders. The median distribution of children to adults in the IPT register implementation period was 2 (range: 1–9). Following the introduction of the IPT register, clinic personnel reported informally that it had been simple and easy to implement.

**Table 2:** Summary of eligible child contacts^1^ identified before and after the implementation of an IPT register in a clinic in Cape Town, South Africa.

	Prior to IPT register	After IPT register implementation
Adult TB cases^2^	79	79
Child contacts identified fromconventional source	24^3^	39^4^
Child contacts documented to havestarted on IPT	4 (17%)^5^	54 (138%)^6^
All child contacts identified peradult TB case,	0.3^7^	0.7^8^
(95% confidence interval)	(0.2–0.4)	(0.6–0.8)

IPT = Isoniazid prophylaxis therapy, TB = tuberculosis.

1 Children less than five years of age exposed to a bacteriologically confirmed TB case (child contacts diagnosed with active disease excluded).

2 Bacteriologically confirmed and not on a drug-resistant treatment regimen.

3 Identified from TB register and TB case folders.

4 Identified from TB register and TB case folders.

5 Identified from the TB register, TB case folders and individual child folders.

6 Identified from the IPT register.

7 Proportion of the child TB contacts identified from the TB register and TB case folders as a proportion of the number of adult TB cases.

8 Proportion of the child TB contacts identified from the TB register, TB case folders and IPT register as a proportion of the number of adult TB cases.

Adherence in the post-register period was estimated by subtracting the last and first treatment dispensing dates recorded (assuming a 30 day calendar month). Conventional rounding practices were followed and if only one date was recorded, the child was classified to have completed treatment for one month. No pill count data was available. Retrospective review of the individual child clinic folders of the children recorded in the IPT register, did not reveal any history of IPT given in 32% (n = 17), while only one month of IPT was recorded in most children (65%, n = 35) and two and five months recorded for two children, respectively. Based on the dates recorded in the IPT register, six months of IPT was completed in 37% (n = 20) of children followed by a course of one month in 28% (n = 15), two months in 28% (n = 15) and three months in 7% (n = 4).

A review of the TB treatment register of the clinic for the period May 2011 to August 2012 did not identify any of the children recorded in the IPT register, as having started TB treatment during this period.

## Discussion

We have shown that using a specific IPT register to guide the delivery of IPT in children, leads to the documentation of more TB-exposed children per adult TB case, and that more of the identified children were recorded as having started IPT. In addition, it was considerably easier to access information on child contacts after the implementation of the register, due to the standard recording of a minimum set of indicators. Previously, extracting the relevant information from various files was laborious and time-consuming.

To our knowledge, this is the only reported evaluation of the WHO recommendation of using an IPT register to facilitate IPT delivery in an African setting. IPT delivery is a challenge in many resource restrained high-burden settings [6], [7], [8], [9], [13], [14] and there are many factors contributing to this complex challenge. Moving the current WHO and SANTP policy into practice, will require a comprehensive approach to address the barriers that prevent IPT delivery to at-risk children from high-burden communities.

Given the historic stigma associated with TB, treating an otherwise healthy child in a TB treatment room might be a barrier to preventive care and moving these children to the care of the routine paediatric nurse might improve treatment adherence and outcomes. Future research could evaluate additional roll-out and adherence support measures including electronic tools, treatment reminders, dedicated treatment supervision and an in-depth evaluation of the knowledge, attitude and practices concerning IPT in both health care providers and the parents/caregivers of children [15]. A cost-benefit analysis of a large scale IPT register implementation could provide valuable direction in terms of the potential benefit of implementing this initiative. In addition to such health systems strengthening, both healthcare providers and TB exposed families should be educated to ensure commitment to post-exposure TB prevention in children. Finally, an official inclusion of IPT reporting indicators into the routine TB programme, similar to existing TB case reporting is likely to increase IPT prioritisation among routine health care workers, if used in combination with routine IPT recording.

We note that this was a small study completed in a clinic with well-functioning TB services and a strong history of participation in TB research, following a change in the routine algorithm of managing TB exposed children less than five years of age. We did not have access to data describing the disease outcomes of any screening procedures done prior to starting IPT and could not report on incident TB, following the pre-register period. In addition, the observed change in recommended local contact screening algorithms introduced with the IPT register could therefore have contributed to the observed improvements that we described. This study was operational in nature using routine data and it was not feasible to evaluate the intervention with a before-and-after study design at this stage; the use of routine data does however raise questions about data accuracy. Despite these limitations, the magnitude of improvement observed suggests that an IPT register could play a valuable role in improving both IPT delivery and potentially, TB case detection in young children in similar settings.

In conclusion, this study has demonstrated a substantial increase in the number of identified child TB contacts and in the proportion of those children who initiated IPT. We therefore suggest that IPT registers be evaluated on a larger operational scale in other settings and circumstances to confirm their value in managing child TB contacts.

## Supporting Information

Figure S1 **Example of the data collection sheet used as IPT register.**
(TIF)
