# Supplementary figures and images for: Does an Isoniazid Prophylaxis Register Improve Tuberculosis Contact Management in South African Children?
Source: PLoS One. 2013 Dec 10;8(12):e80803. doi: 10.1371/journal.pone.0080803 (PMC3858233; doi:10.1371/journal.pone.0080803)

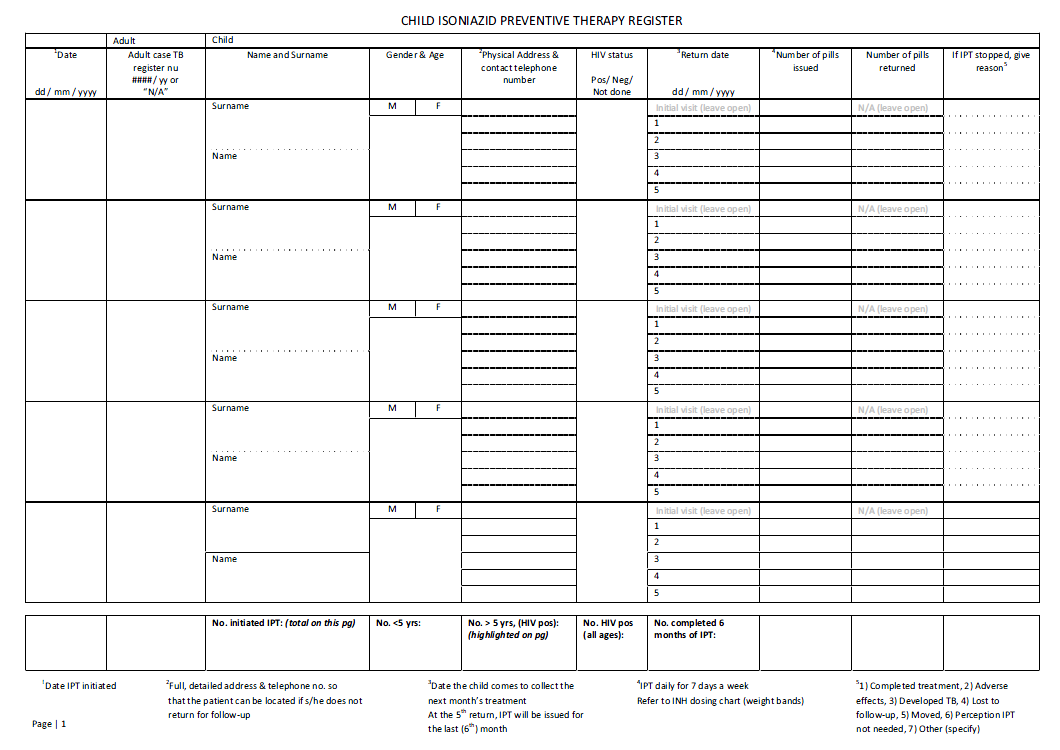

Supplement: Figure S1 — Example of the data collection sheet used as IPT register. (TIF) [file pone.0080803.s001.tif]
